# Possible important roles of galectins in the healing of human fetal membranes

**DOI:** 10.3389/fendo.2022.941029

**Published:** 2022-08-09

**Authors:** Jia-Le Chen, Yu Chen, De-Xiang Xu, Dao-Zhen Chen

**Affiliations:** ^1^ The School of Public Health, Anhui Medical University, Hefei, China; ^2^ Research Institute for Reproductive Health and Genetic Diseases, Wuxi Maternity and Child Health Care Hospital, Wuxi, China; ^3^ Department of Laboratory, Haidong No.2 People’s Hospital, Haidong, China

**Keywords:** amniotic epithelial cells, amniotic mesenchymal cells, galectin, macrophages, fetal membrane, wound healing

## Abstract

The fetal membranes healing is a complex and dynamic process of replacing devitalized and missing cellular structures and tissue layers. Multiple cells and extracellular matrices, and cell differentiation, migration and proliferation may participate in restoring the integrity of damaged tissue, however this process still remains unclear. Therefore, there is a need to identify and integrate new ideas and methods to design a more effective dressing to accelerate fetal membrane healing. This review explores the function and role of galectins in the inflammatory, epithelial mesenchymal transition, proliferative migration, and remodeling phases of fetal membrane healing. In conclusion, the preliminary findings are promising. Research on amnion regeneration is expected to provide insight into potential treatment strategies for premature rupture of membranes.

## Introduction

Premature rupture of the fetal membranes (PROM) is frequently linked to poor postnatal outcomes (1, 2). The membranes may separate and result in tissue injury and amniotic fluid loss (3). The fetal membranes consist of the amnion and chorionic villus (4, 5). The amnion layer is extremely elastic, as it supports fetal development stretches and stresses as well as amniotic fluid buildup throughout pregnancy (6). The chorion layer, which is thicker and stiffer than the amnion, slips over the amnion layer (7). The amnion exhibits striking regenerative potential in few but exciting clinical trials. Furthermore, preclinical wound healing of the fetal membrane has been already reported by the *in vitro* experiments (amniotic epithelial cells, puncture model of the fetal membrane) (8, 9) and the *in vivo* system (mice, rabbits) (9, 10), as described in **Table 1**. However, the key molecular mechanism of fetal membranes to heal wounds after spontaneous or iatrogenic membrane rupture still remains unknown. Previous researches have demonstrated that galectins are a key player in the membrane healing process such as the healing of skin and cornea (11, 12), nonetheless, although galectins are overexpressed on fetal membranes in patients with PROM (13, 14), their function in fetal membrane healing is still unidentified. In this review, we will present the current knowledge on fetal membrane wound healing and discuss the possible role galectins may play in for the development of clinical solution to improve healing of the fetal membranes.

**Table 1:** Representative Preclinical studies of fetal membranes healing.

Author	Model	Highlight	Key molecules	Title of reference
Sopher D.	rat/in vivo	21G needles: resulted in a proliferation of amnion mesenchymal cells at the edge of the amnion within 24 h.	EMT and migration: amnion mesenchymal cells	The response of rat fetal membranes to injury.
Nicole Ochsenbein-Kölble	AEC Culture/in vitro	Cultures showed good survival for 14 days. Increased cellularity, survival and proliferations were observed.	Proliferation: EGF↑, insulin↑, transferrin↑	Inducing proliferation of human amnion epithelial and mesenchymal cells for prospective engineering of membrane repair.
Haruta Mogami	mouse/in vivo	Small ruptures of the fetal membrane closed within 72 h whereas healing of large ruptures was only 40%.	Inflammation: IL-1β↑, TNF↑, Arg1^ + ^macrophages↑	Healing of Preterm Ruptured Fetal Membranes.
Haruta Mogami	mouse/in vivo	26 or 20 G needles: Collagen type 1 injection: 90% healed within 72 h; PBS: 40% healed.	Adhesion and migration: integrins, collagen receptors discoidin domain receptors	Collagen Type 1 Accelerates Healing of Ruptured Fetal Membranes.
Ah-youngLee	amniotic pore culture/in vitro	20 G needles: 100% healed; 26G needles:40% healed.	EMT: CD49↑,TRA 1-60↑,SSEA-4↑, OCT-4↑, Nanog↑	Spontaneous healing of human amnion in the premature rupture of membrane model.
Lauren Richardson	AEC Culture/in vitro	Healing progress: proliferation, migration, transition, and self-renewal.	Inflammation: IL-8↑, EMT/MET: vimentin/CK-18 ratio, E-cadherin↑, N-cadherin↑, Collagen Type 1 ↑	Proliferative, Migratory, and Transition Properties Reveal Metastate of Human Amnion Cells.
R Devlieger	fetoscopic/in vivo	Collagen plugging of the fetoscopic access port sites in sheep resulted in functionally effective sealing of the fetal membranes.	Remodling: MMP-2↑, MMP-9↑, and TIMPs↓	Matrix metalloproteinases-2 and -9 and their endogenous tissue inhibitors in tissue remodeling after sealing of the fetal membranes in a sheep model of fetoscopic surgery.

## Healing of the fetal membranes

Generally, the amnion consists of two types of cells: superficial epithelial cells and inferior mesenchymal cells. As is reported, the interstitial collagen (types I, III and V) is produced by amniotic mesenchymal cells to maintain the mechanical integrity (15). In some cases, such as amniocentesis, the ruptured membranes could spontaneously “reseal” (16, 17). Remarkably, rats were the first used to make histological investigations of the healing process in embryonic membranes. Sopher (18) demonstrated that puncturing rat gestational sacs on day 15 of pregnancy with a 21-gauge needle resulted in the proliferation of amnion mesenchymal cells within 24 hours. Similarly, *via* both primary human amnion cells in culture and a aseptic puncture model in mice, Mogami (9) demonstrated that tiny rupture of fetal membrane in the second trimester of pregnancy (0.47 mm) healed successfully in the mice model. Furthermore, after undergoing collagen transplantation to treat spontaneous PPROM patients at 16.5 weeks of pregnancy, the patients successfully delivered boys with an Apgar score of 6-7 at 30 weeks and 3 days, and the boys grew up to be healthy and active adults (19). Despite this, only a few explants and the interactions between the membranes and the sealant were investigated *in vivo* throughout the complete gestational age. Since the healing of cultured fetal membrane explants may take a long period, *in vitro* tissue explants become increasingly sick with time, which may limit their ability to repair (20).

Wound healing is a process that restores damaged skin to maintain tissue homeostasis. Adult epidermal wound healing is divided into four main stages: hemostasis, inflammation, proliferation and migration, and regression, remodeling and healing. (21) Fetal tissue healing is much simpler compared to adult tissue (21), the process of amnion membrane tissue rupture is not accompanied by vascular damage, granulation tissue is usually not formed, and inflammation is suppressed to a minimum. The healing of fetal membranes may be initiated by rupture signal activation involved in the recruitment of inflammatory cells (22), while the vascular damage in the chorionic villus may also contribute to injury responses in the fetal membranes (23). In a mouse model of sterile PROM, macrophages are recruited to the site of injury. A well-tissue local inflammatory response induces epithelial mesenchymal transition of amniotic epithelial cells, which accelerates cell migration and healing of the amnion (24). Therefore, it is hypothesized that the healing of fetal membranes (especially healing of amnion membrane) may consist of four stages: ① inflammation, ② epithelial mesenchymal transition EMT, ③ proliferation and migration (mostly migration) (20), and ④ remodeling (it is difficult for fetal membrane to heal completely without normal intervention). It is critical at this stage in the repair of the fetal membrane to increase the potential for epithelial cell migration across the wound.

## The role of galectin in the healing of fetal membranes

Galectins, which are carbohydrate-binding proteins that bind to complex carbohydrates on the cell surface and in the extracellular matrix, can decipher this information and govern the interaction between cells or between cells and the ECM (25–27). Over the last few decades, galectins have been identified as crucial for implantation and pregnancy maintenance (28, 29). In a growing number of studies, their role in trophoblast cell function and placental development has been revealed (30). In addition, evidence show that they play key roles in the control of fetal-maternal immunological tolerance and angiogenesis (31). Furthermore, it has also been recognized for its important function in healing. We intend to synthesize current understanding about galectins in the healing of fetal membrane in this review. Galectin family members may activate diverse signal transduction pathways and govern different biological processes, depending on their position within the cell. In the maternal-fetal interface, it is known that 9 subtypes of galectins are substantially expressed, such as galectin-1、-3、-7、-8、-9、-13、-14、-15、-16 (32). Studies demonstrating that galectins are involved in a variety of key cellular responses, including cell-cell and cell ECM interactions, immunological tolerance, and the anti-regulation of the inflammatory response (33–35). Galectin-1 is highly involved in the regulation of the immune response. The presence of significant galectin-1 on the surface of placental mesenchymal stromal cells derived from chorionic villi in early pregnancy, as well as the presence of this galectin in exosomes, opens up a new avenue for galectin-1 to function as a key signal molecule in the placental secretory group (36). Evidence suggests that galectin-1 can exist in integrin subunits, as well as interact with ECM glycoproteins such as fetal fibronectin and laminin, which may have an effect on cell invasion (37). In line with this, human amniotic epithelium expresses galectin-1, galectin-3, galectin-7 and galectin-8, and galectin-3 is significantly increased in the amniotic epithelium of patients with chorioamnionitis (14). The theory of the healing progress may improve the prognosis of premature rupture of membranes.

## Inflammation

**Figure 1:**
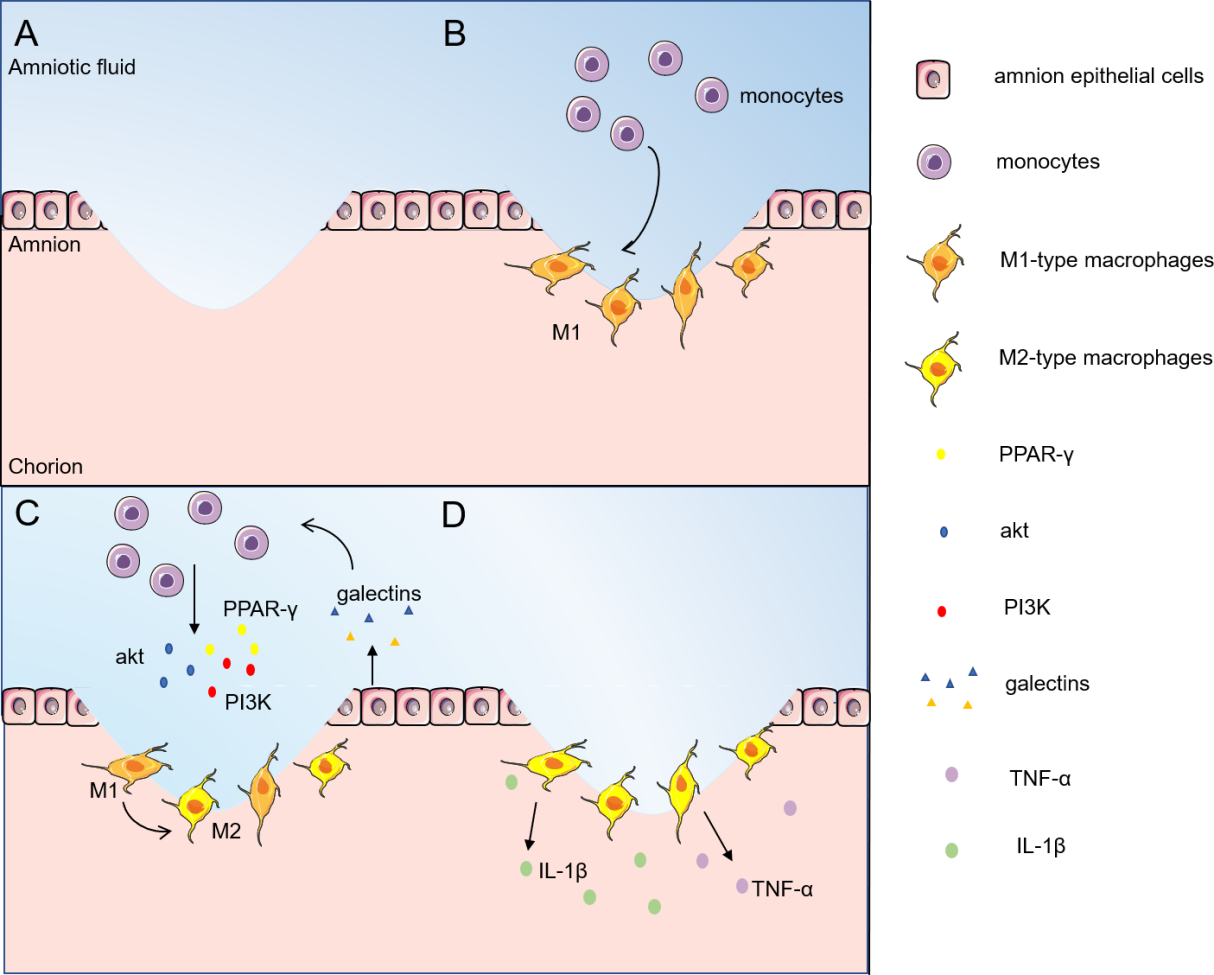
Inflammatory phase of fetal/amnion membrane healing. The inflammatory response caused by fetal membrane injury provides the possibility to initiate the healing of fetal membranes. **(A)** The fetal membranes are ruptured by external infection or mechanical injury; **(B)** Monocytes are first recruited from amniotic fluid to the site of injury and transformed into macrophages; **(C)** Galectins stimulate monocytes to enhance the expression levels of Akt, PI3K and PPAR-γ, and turn on the M2 polarization of macrophages; **(D)** M2-type macrophages release TNF-α, IL-1β and other cytokines to promote fetal membrane healing. M2-type macrophages release cytokines such as TNF-α and IL-1β to promote the healing of amnion membranes. akt, protein kinase B; PI3K, phosphatidylinositol-3 kinase; PPAR-γ, peroxisome proliferator-activated receptor-gamma; TNF-α, tumor necrosis factor-α; IL- 1β, interleukin-1β.

As can be seen in **Figure 1**, the inflammatory response caused by fetal membrane injury provides the possibility of initiation of healing of the fetal membrane (38, 39). In wound healing such as epidermal neutrophils are among the first leukocytes recruited during inflammation, migrating from the damaged vascular system. Monocytes are usually recruited to wounds later than neutrophils; once there, they differentiate into macrophages, which remove debris as well as dying neutrophils by phagocytosis (40). However, studies have observed that only macrophages accumulate around aseptically ruptured amniotic membranes. In contrast to the typical epidermal wound healing process in adults, migration of neutrophils was rarely observed (9). This may be related to the aseptic nature of the absence of infection and inflammatory stimuli. M2-dominant macrophages are recruited from the amniotic fluid to the site of injury and release cytokines such as TNF-α and IL-1β to promote healing of the fetal membranes (9). Therefore, M2 macrophages may play a central role in the inflammatory process of fetal membranes.

Among all family members, galectin-1, galectin-3 and galectin-9 are highly expressed in immune cells. Peritoneal macrophages from galectin-3-deficient mice showed higher levels of apoptosis compared to cells from wild-type mice, suggesting that galectins may also protect macrophages from cell death (41). Galectin-1 does not affect macrophage survival and also stimulates the ERK1/2 signaling pathway to regulate macrophage activity by promoting arginase activity (42) and PGE2 production (43, 44). In addition, galectin-1 has been shown to promote M2 macrophage phenotypic polarization, tissue repair and regeneration (45). In the presence of galectin-3 inhibitors, M2 markers (mannose receptors) are downregulated and M1 markers (iNOS) are upregulated on RAW264.7 macrophages, and this phenotypic skew suggests that galectin-3 promotes macrophage polarization toward the M2 phenotype (46). THP-1 monocytes co-cultured with human-galectin-9-expressing porcine kidney epithelial cells turned on the M2 differentiation program while decreasing the M1 differentiation program by enhancing the phosphorylation levels of Akt and PI3K as well as the expression level of PPAR-γ (47). LPS regulates macrophage M1/M2 subtype polarization through the galectin-9/Tim-3 signaling pathway, and upregulation of galectin-9 polarizes macrophages to M2 (48). Galectins can be found to promote macrophage survival and stimulate macrophage M2 phenotype polarization to generate an inflammatory response and initiate fetal membrane healing. These findings suggest that galectins could be selected as a potential therapeutic target for fetal membrane rupture to modulate the immune system and promote tissue regeneration.

## Epithelial mesenchymal transition (EMT)

**Figure 2:**
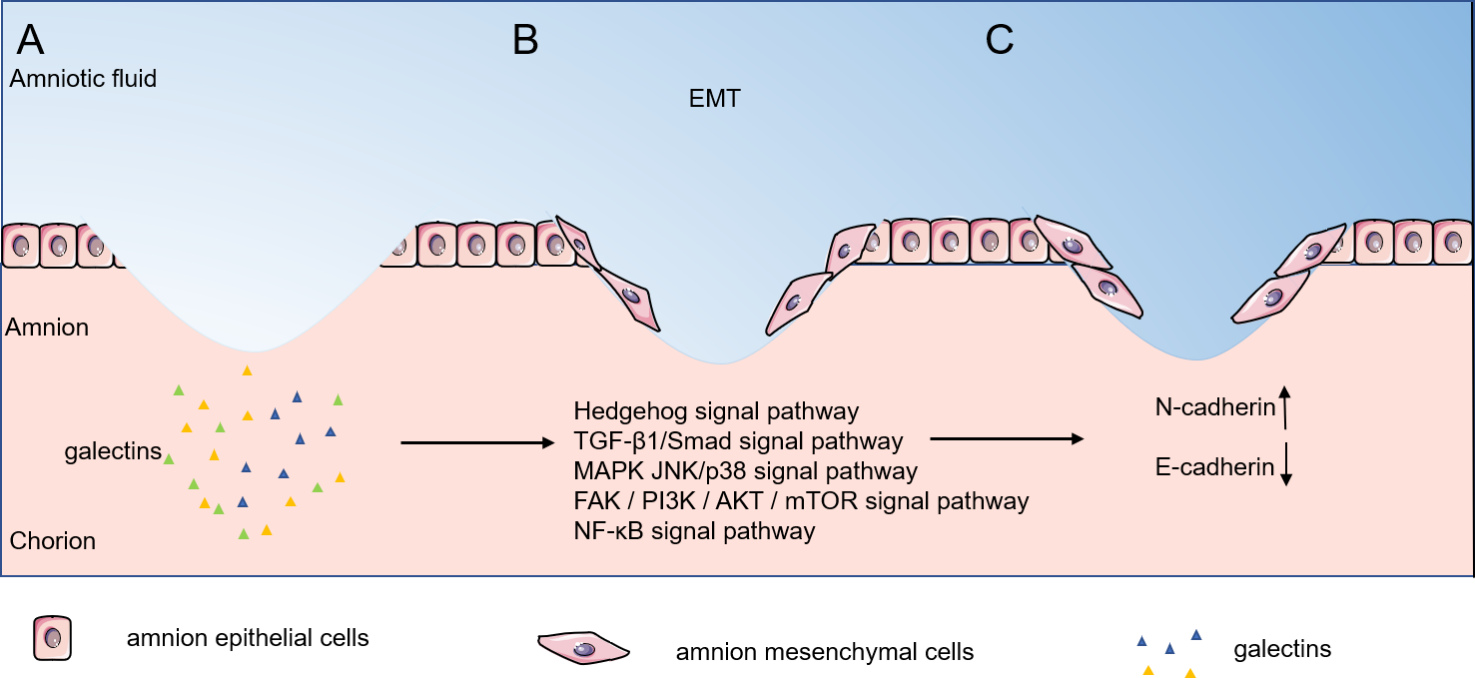
Epithelial-mesenchymal transition phase of fetal/amnion membrane healing. Expression of inflammatory factors after amnion membrane rupture can promote epithelial- mesenchymal transition, and EMT can accelerate cell migration, thus accelerating wound closure. **(A–C)** Galectins stimulate amnion epithelial cells through the Hedgehog signal pathway, TGF-β1/Smad signal pathway, MAPK JNK/p38 signal pathway, FAK / PI3K / AKT / mTOR signal pathway and NF-κB signal pathway stimulated the transformation of amnion epithelial cells into amnion mesenchymal cells. EMT, epithelial-mesenchymal transition.

As illustrated in **Figure 2**, expression of inflammatory factors after fetal membrane rupture can promote the development of epithelial-mesenchymal transition. Interleukins, TNF-α, and IFN-γ are able to induce EMT and the ability of epithelial cells to transform into myo- mesenchymal stem cells through different signaling pathways. In pPROM mice, waveform protein-positive cells can be observed scattered in the epithelial layer of the ruptured mouse amnion, indicating the occurrence of EMT *in vivo*. EMT is known to accelerate cell migration and thus wound closure.

Despite few research on the participation of the galectin family in EMT, the studies in tumor cells may provide possibility of its involvement in fetal membrane EMT. In galectin-1 overexpressing suppressor T cells (TS cells), galectin-1 promotes EMT by upregulating the expression of N-cadherin and downregulating the expression of E-cadherin, inducing TS cell migration and invasion (49). In gastric cancer fibroblasts (CAF), galectin-1 stimulated high expression by upregulating β1-integrin (50) and induced EMT through the Hedgehog signaling pathway (51), which promoted migration and invasion of cancer cells. Activation of TGF-β by galectin-3 induces EMT in lung cancer A549 cells (52). In fibroblasts, galectin-1 induced EMT through the TGF-β1/Smad signaling pathway thereby promoting invasion and metastasis of gastric cancer cells (53). Galectin-1 induces epithelial mesenchymal transition (EMT) in human ovarian cancer cells through activation of the MAPK JNK/p38 signaling pathway (54). In human prostate cancer transplants, galectin-4 binding to receptor tyrosine kinases activated the expression of phosphorylated ERK, phosphorylated Akt and Twist and decreased E-cadherin expression, thereby promoting EMT (55). Similarly, in upper urothelial migratory cell carcinoma, galectin-1 promoted EMT by activating the FAK/PI3K/AKT/mTOR pathway significantly reduces MMP-2 and MMP-9 levels and promotes EMT (56). Furthermore, in human pancreatic cancer, galectin-1 triggers EMT through NF-κB transcriptional regulation and induces significant overexpression of invasion and migration-related genes including MMP1, S100A7, and ankyrin-3, thus playing a major metastatic invasive role (57). Taken together, we hypothesize that galectins can induce matrix metalloproteinase production through multiple signaling pathways thereby promoting cell migration and achieving wound healing.

## Proliferation and migration

**Figure 3:**
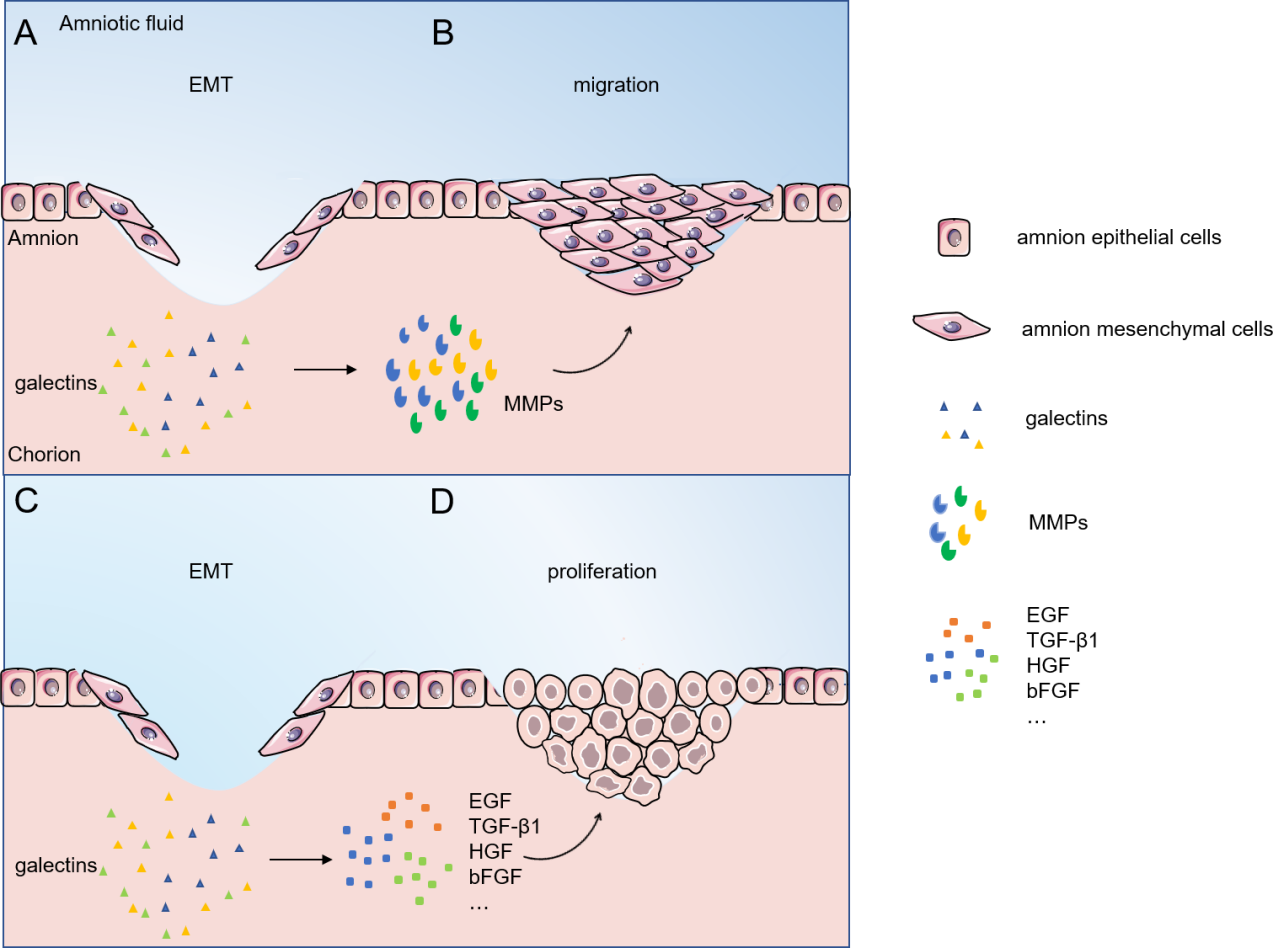
Migratory proliferative phase of fetal/amnion membrane healing. **(A, B)** Galectins regulate cells migration by interacting with MMPs; **(C, D)** Galectins regulate cells proliferation by interacting with EGF, TGF-β1, HGF, bFGF and other proteins. Abbreviations: EMT, epithelial-mesenchymal transition; MMPs, matrix metalloproteinases; EGF, epidermal growth factor ; TGF-β1, transforming growth-factor-β1; HGF, hepatocyte growth factor; bFGF, basic fibroblast growth factor.

**Figure 3** shows a hypnosis that, after EMT occurs, it induces cell proliferation, synthesis of collagen, hyaluronic acid, etc., and extracellular matrix (ECM) formation. HAESCs promote wound healing by facilitating the migration and proliferation of keratinocytes *via* ERK, JNK and AKT signaling pathways (58). Growth factors are among the most important proteins for human cell proliferation. Proteins such as epidermal growth factor (EGF), transforming growth factor β1 (TGF-β1), hepatocyte growth factor (HGF), and basic fibroblast growth factor (bFGF) are structurally active in amniotic cells and promote amniotic cell growth (59). Intestinal epithelial cells lacking galectin-9 expression exhibit an attenuated proliferative response in the presence of regenerative stimuli (60). Galectin-1 can interact with cell surface growth factors, stimulate their phosphorylation, and affect the proliferation of umbilical vein endothelial cells (61). In addition, Galectin-1 was found affecting the development of mouse trophoblast stem cells *in vitro* and promoting the expression of the Matrix metalloproteinase (MMP) and the TGF-β1 (49). In epithelial cells, galectin-7 regulates the proliferation and differentiation of keratin-forming cells through the JNK1-miR-203-p63 pathway (62). On the other hand, there is evidence that galectin-3 regulates the Smad2/3 signaling pathway through interaction with TGF-β1 protein, which in turn regulates the proliferation and migration of human pulmonary artery smooth muscle cells. Moreover, TGF-β1 and galectin-3 can mutually regulate the expression levels of proteins and mRNAs (63). Studies have showed that active collagen synthesis, matrix remodeling occurs in the healing membrane after a major rupture of the fetal membrane (9). Glycan modification may affect proliferative signaling due to transcriptional or epigenetic regulation of glycan-modifying enzymes and altered expression of glycan-binding proteins (64). Galectin-3 deficiency in Mst1-TG mice leads to dysregulation of signaling related to extracellular matrix remodeling and collagen formation (65). Epithelial cell proliferation is significantly decreased, collagen-I accumulation is decreased, and alpha smooth muscle actin expression is downregulated in galectin-9-deficient mice. Galectin-9 promotes extracellular matrix synthesis and collagen accumulation *via* TGF-β-induced MAPK/ERK, TAK1/JNK, and PI3K/AKT signaling pathways (66). Galectin-8 stimulates TGF-β1 *via* β1-integrin/FAK pathways to enhance type I collagen, fibronectin FN and connective tissue growth factor CTGF protein levels in human gingival fibroblasts (67).

Studies have shown that cells closest to the wound preferentially migrate, while cells far from the wound preferentially proliferate (68). Galectins significantly affect this marker by regulating metastasis-related events. Indeed, galectin-3 has been identified as a glycoprotein associated with cell motility (8). Galectin-3 binds to the cell adhesion glycoprotein CD146 to promote cytokine secretion and mediated endothelial cell migration (69). On the other hand, galectin-3-silenced HTR-8/SVneo cells showed a significant reduction in migration and invasion, low-level expression of MMP-2 and MMP-9, and a massive reduction in integrin β1 (70). Microarray analysis showed that treatment with galectin-1 induced upregulation of genes encoding matrix metalloproteinases MMP-1, MMP-10, MMP-12 and tissue fibrinogen activator in dendritic cells, which was associated with increased migratory activity of these antigen-presenting cells (71). In addition, transfection of galectin-4cDNA into placental trophoblast cells induced enhanced Akt phosphorylation upregulated the expression of MMP-9 and N-Cadherin, promoting trophoblast migration and invasion (72).

## Remodeling

In contrast, during the remodeling phase, as inflammation gradually decreases, the proliferative process is downregulated to avoid excessive tissue proliferation and is gradually replaced by the initiation of collagen rearrangement (38, 39). During the remodeling phase, most of the endothelial cells, macrophages and fibroblasts undergo apoptosis and human amniotic mesenchymal cells (AMC) undergo MET to re-differentiate into amniocytes and form the fetal membrane tissue together with the rearranged collagen and extracellular matrix. Amniocytes dynamically switch between epithelial and mesenchymal states to maintain amniotic membrane integrity and repair membrane damage, as well as to respond to inflammation and mechanical injury to protect the fetus until delivery (8, 73). During this process, collagen is rearranged by the action of matrix metalloproteinases, gradually forming an ordered matrix structure, and the major component changes from type III to type I collagen. However, the role of galectins in amniotic tissue remodeling has not been studied yet.

Studies of the role of galectins in wound healing have revealed that galectin-3 promotes re-epithelialization of corneal, intestinal and skin wounds; galectin-7 promotes re-epithelialization of corneal, skin, kidney and uterine wounds; and galectin-2 and galectin-4 promote re-epithelialization of intestinal wounds. Galectin-3 is present on the cell surface, within ECM, and in the cytoplasm. Galectin-3 influences cell-matrix interaction by binding to the ECM and cell surface glycosylated counter receptors (e.g., growth factor receptors, integrins, certain isoforms of laminin, fibronectin and vitronectin). Furthermore, Galectin-3 in the nucleus of cells may influence cell-matrix interactions indirectly by its effect on the expression of well-known cell adhesion molecules (e.g., α6β1 and α4β7 integrins) and cytokines (e.g., IL-1) (12). Galectin-3 is present in high density at sites of corneal epithelial cell-matrix adhesion (74), an ideal placement for influence on cell-matrix interactions and cell migration. In a recent study, using galectin3^−/−^ mice and cells isolated from these mice, Liu et al. (75)demonstrated that the absence of galectin-3 impairs keratinocyte migration and skin wound re-epithelialization. Interestingly, in this study, the promigratory function of the lectin was attributed to cytosolic galectin-3, and, therefore, is likely to be carbohydrate independent. Lagana et al. (76) demonstrated that galectin-3 interactions with N-acetylglucosaminyltransferase V (GnT-V)-modified N-glycans on mammary carcinoma cell surface support α5β1 integrin activation and cell motility. Noorjahan et al. (77) have demonstrated that galectin-3 by interacting with GnT-V-modified complex N-glycans, activates α_3_β_1_-integrin-Rac1 signaling to induce formation of lamellipodia in epithelial cells, and, this in turn, promotes cell migration and re-epithelialization of wounds.

## Summary & outlook

We hypothesize that the amnion is capable of healing based on previous research. Wound management agents should actively regulate the fetal membrane (especially healing of amnion membrane) phase and several strategies exist that can support the maintaining of fetal membrane structural integrity. In this review, we summarize the role of galectins in fetal/amniotic membrane healing. During the various stages of amniotic membrane healing, galectins positively influence amniotic membrane wound healing through various activities, including macrophage polarization, cell proliferation migration, collagen synthesis, and ECM remodeling. *In vivo* and *in vitro* data suggest that galectins can aid amniotic membrane healing by modulating inflammation and supporting cell proliferation. However, additional data from human systemic studies are needed to fully elucidate the mechanism of action of galectins and support their efficacy in fetal membrane healing.

## Author contributions

D-ZC and D-XX conceived the central idea. J-LC screened the literature and wrote the initial draft of the study. The remaining authors contributed to refining the ideas, carrying out additional analyses, and finalizing this study. All authors contributed to editorial changes in the manuscript. All authors read and approved the final manuscript.

## Funding

This research was funded by Development and Demonstration Program of Wuxi (grant No. N20192004), Key Research and Development Program of Jiangsu Province (grant No. BE2015617), and Anhui Provincial Education Department Scientific Research Project (Postgraduate Innovation Research and Practice Program of Anhui Medical University)(YJS20210285).

## Conflict of interest

The authors declare that the research was conducted in the absence of any commercial or financial relationships that could be construed as a potential conflict of interest.

## Publisher’s note

All claims expressed in this article are solely those of the authors and do not necessarily represent those of their affiliated organizations, or those of the publisher, the editors and the reviewers. Any product that may be evaluated in this article, or claim that may be made by its manufacturer, is not guaranteed or endorsed by the publisher.
